# GEB-YOLO: a novel algorithm for enhanced and efficient detection of foreign objects in power transmission lines

**DOI:** 10.1038/s41598-024-64991-9

**Published:** 2024-07-09

**Authors:** Jiangpeng Zheng, Hao Liu, Qiuting He, Jinfu Hu

**Affiliations:** Key & Core Technology Innovation Institute of the Greater Bay Area, Guangzhou, 510535 China

**Keywords:** Foreign objects detection, GhostConv, Feature fusion, BiFPN, YOLOv8n, Energy grids and networks, Power distribution

## Abstract

Detecting foreign objects in power transmission lines is essential for mitigating safety risks and maintaining line stability. Practical detection, however, presents challenges including varied target sizes, intricate backgrounds, and large model weights. To address these issues, this study introduces an innovative GEB-YOLO model, which balances detection performance and quantification. Firstly, the algorithm features a lightweight architecture, achieved by merging the GhostConv network with the advanced YOLOv8 model. This integration considerably lowers computational demands and parameters through streamlined linear operations. Secondly, this paper proposes a novel EC2f mechanism, a groundbreaking feature that bolsters the model’s information extraction capabilities. It enhances the relationship between weights and channels via one-dimensional convolution. Lastly, the BiFPN mechanism is employed to improve the model’s processing efficiency for targets of different sizes, utilizing bidirectional connections and swift feature fusion for normalization. Experimental results indicate the model’s superiority over existing models in precision and mAP, showing improvements of 3.7 and 6.8%, respectively. Crucially, the model’s parameters and FLOPs have been reduced by 10.0 and 7.4%, leading to a model that is both lighter and more efficient. These advancements offer invaluable insights for applying laser technology in detecting foreign objects, contributing significantly to both theory and practice.

## Introduction

Power system stability and security heavily rely on the timely identification of extraneous objects on transmission lines^1^. T These lines, spanning diverse and challenging terrains such as mountains, urban areas, and construction zones, are particularly prone to damage and present maintenance complexities^2^. In response to escalating electricity demands, nations are expanding their networks of high-voltage transmission lines^3^. This expansion necessitates substantial investments from power companies in both line inspection and maintenance^4^. External objects like kites, balloons, and bird nests are common causes of line faults, underscoring the urgency of advanced maintenance approaches. Developing efficient detection techniques for these objects on transmission lines is crucial to maintain the integrity and reliability of power systems^5^.

Initially, foreign object detection on transmission lines was predominantly manual. Traditional foot patrols^6^, for instance, required teams of two to inspect lines by walking from one tower to the next. However, this approach was often hindered by challenging terrain and adverse weather conditions, posing safety risks to personnel. An alternative involved using helicopters equipped with high-definition cameras and advanced infrared thermal imagers for aerial surveillance^7^. While this method enhanced both efficiency and safety, it was costly, required specialized operators, and lacked flexibility. In light of these limitations, computer vision-based detection methods have emerged as a promising solution.

Traditional computer vision techniques largely depend on manually crafted feature extraction methods^8^. For example, Yao et al.^9^ proposed utilizing color and texture attributes from expanded areas, combined with radial basis function kernels in support vector machines, for categorizing transmission lines. In a similar vein, Lin and colleagues^10^ enhanced image edge detection for foreign objects on high-voltage lines, using an improved Canny operator. Nevertheless, these techniques, reliant on manual feature extraction, struggle in complex environments and lack the capability for real-time detection, thus limiting their practicality.

Deep learning has markedly advanced object detection in terms of accuracy and speed, surpassing traditional meth-ods^11,12^. These techniques are categorized into two types: two-stage methods^13–16^ and one-stage meth-ods^17–20^. Two-stage algorithms involve candidate region generation followed by object classification and localization. For example, Zhang’s team^21^ enhanced detection accuracy and speed with a novel network based on Fast R-CNN. Similarly, Chen’s team^22^ proposed a remote learning approach using an improved Faster R-CNN, employing. Transfer learning and data augmentation to identify various foreign objects. Additionally, Chen et al.^23^ developed a visual detection method for power transmission line foreign objects using Mask R-CNN, which increased recognition accuracy and reduced sensor usage through image enhancement. The study by Yu et al.^24^ introduced an innovative approach that integrates multi-network feature fusion with a random forest algorithm for detecting foreign objects on transmission lines. This approach innovatively integrates diverse network architectures to enhance detection accuracy. However, it may need further refinement for handling varying environmental conditions and object complexities, as indicated by the research findings. Study by Lu et al.^25^ The presents a novel method for detecting bird nests in high power lines near remote campuses, utilizing a combination of features and a cascade classifier. This paper highlights an innovative approach that significantly improves detection accuracy in challenging environments. While the method shows promising results, it may benefit from further refinement in adapting to diverse environmental conditions and varying nest characteristics. However, these methods are complex and not well-suited for resource-constrained embedded devices. In contrast, one-stage algorithms streamline training and inference, omitting complex region proposals, and often outperform two-stage methods in speed.

One-stage algorithms are renowned for efficiently identifying object categories and precise locations^26–29^. Zongqi’s team^30^, for instance, developed a YOLOv2-based method for detecting foreign objects on power transmission lines. While effective, its accuracy is limited in complex settings. Li’s team^31^ refined the YOLOv3 model by reducing convolution kernels and network parameters, albeit with some loss in accuracy. Li et al.^32^ conducted a notable study on power transmission line foreign object detection, leveraging an improved YOLOv3 model. Their work showcases the model’s deployment on a chip for enhanced practical application. This study stands out for its integration of advanced object detection techniques with real-world utility in power transmission systems. Song’s team^33^ improved a YOLOv4-based model using K-means clustering and DOONMS optimization, which, however, increased the model’s complexity. The YOLOv5 model by Yuan’s team^34^ incorporated an attention mechanism and a layer for small object detection in automated systems, albeit with a risk of overfitting.

Yu’s^35^ team’s YOLOv7 model combined genetic algorithms and spatial depth convolution for better localization accuracy, though at greater computational costs. Meanwhile, Wang’s team^36^ enhanced the YOLOv8m model with a global attention module for improved efficiency.

Given the existing challenges of large model weights and diverse target sizes in detecting foreign objects, this study proposes the GEB-YOLO model, achieving a balance between lightweight design and high performance. First, it integrates the lightweight GhsotConv model with advanced YOLOv8 to create a lightweight network. Secondly, it innovatively designs the EC2f structure to enhance information extraction capabilities. Lastly, it optimizes the PAN structure with bidirectional cross-scale connections and rapid feature fusion to improve processing efficiency for targets of different sizes.

This study is organized as follows: Section “Methodology and materials” elaborates on YOLOv8 and the refined GEB-YOLO model; Section “Experimental design and results” discusses the dataset and experimental outcomes; Sections “Discussion” and “Conclusions” delve into a detailed analysis of the experimental results and address potential limitations.

## Methodology and materials

### YOLOV8n network structure

**Figure 1:**
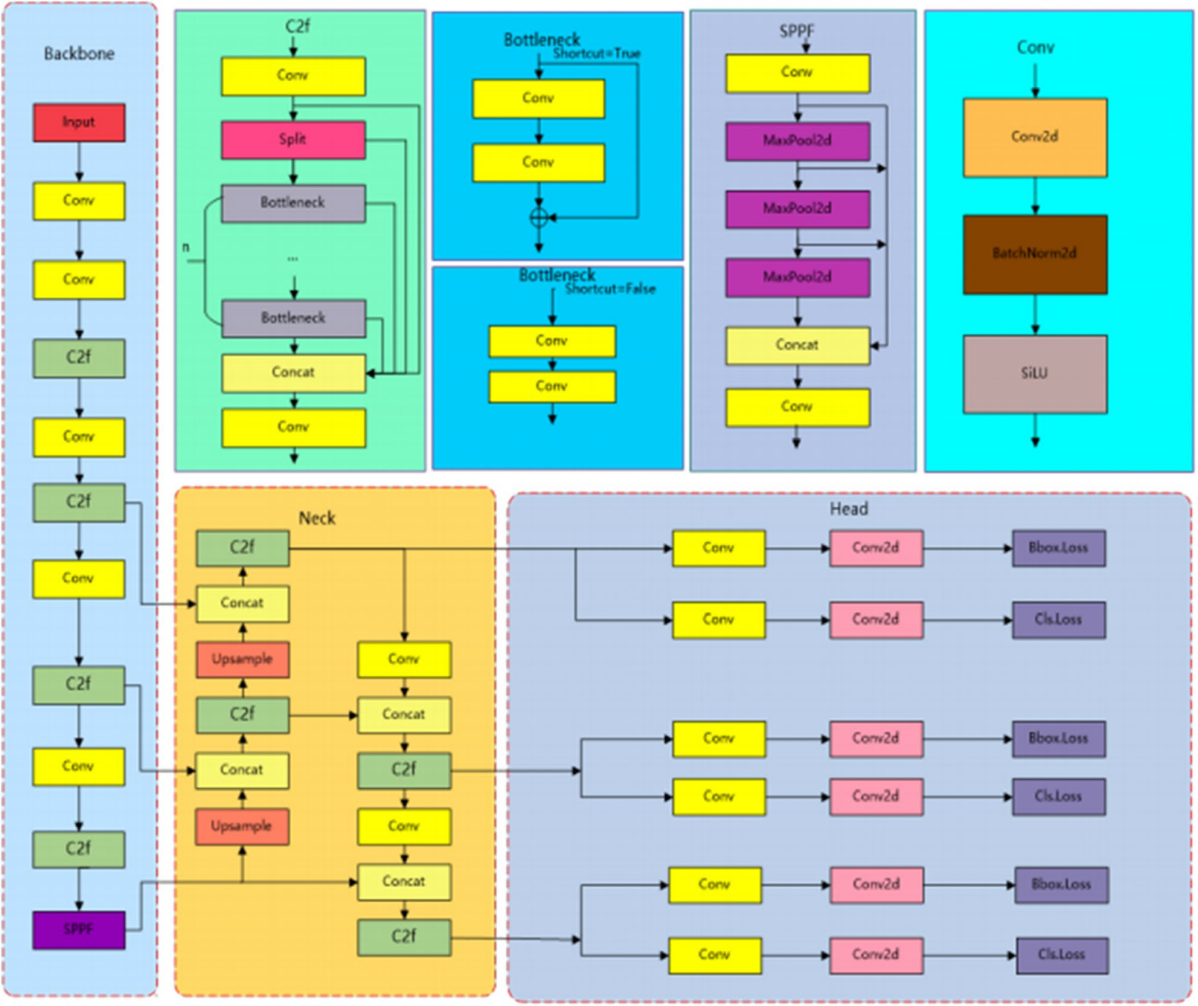
YOLOv8n structural diagram.

YOLOv8, the latest in the YOLO series, is notable for its rapid detection speed and high accuracy. This study employs YOLOv8 for object detection. The model comes in five versions-YOLOv8n, YOLOv8s, YOLOv8m, YOLOv81, and YOLOv8x-each varying in network depth and width. YOLOv8n, selected for its balance of compact size and accuracy, suits our experimental needs. The model’s architecture includes four main components: the Input layer, Backbone network, Neck network, and Head section, as illustrated in Fig. 1.

The Input layer handles multiple image data augmentation processes, including resizing, tone adjustments, and mosaic enhancement. Unlike traditional methods.

YOLOv8n utilizes an anchor-free approach, predicting target centers directly without anchor box offsets.

The Backbone network, integral for feature extraction, includes a Conv module. The C2fmodule introduces extra cross-layer connections, improving gradient flow. The SPPF module employs triple continuous pooling, reducing computations and broadening the receptive field.

The Neck consists of the FPN and PAN. It improves and merges features from different scales. FPN constructs a feature pyramid from convolutional neural network-generated feature maps, integrating them with coarser maps through upsampling. PAN fuses the features from different layers via convolution, maintaining accurate spatial details. This synergy efficiently merges vertical information flow within the network, boosting detection performance.

Lastly, the Head section separates classification from detection. YOLOv8n’s decoupled head design assigns independent detectors to each scale, enabling specialized boundary box predictions at different network layers.

### Enhanced GEB-YOLO network architecture enhancements in ghostConv

**Figure 2:**
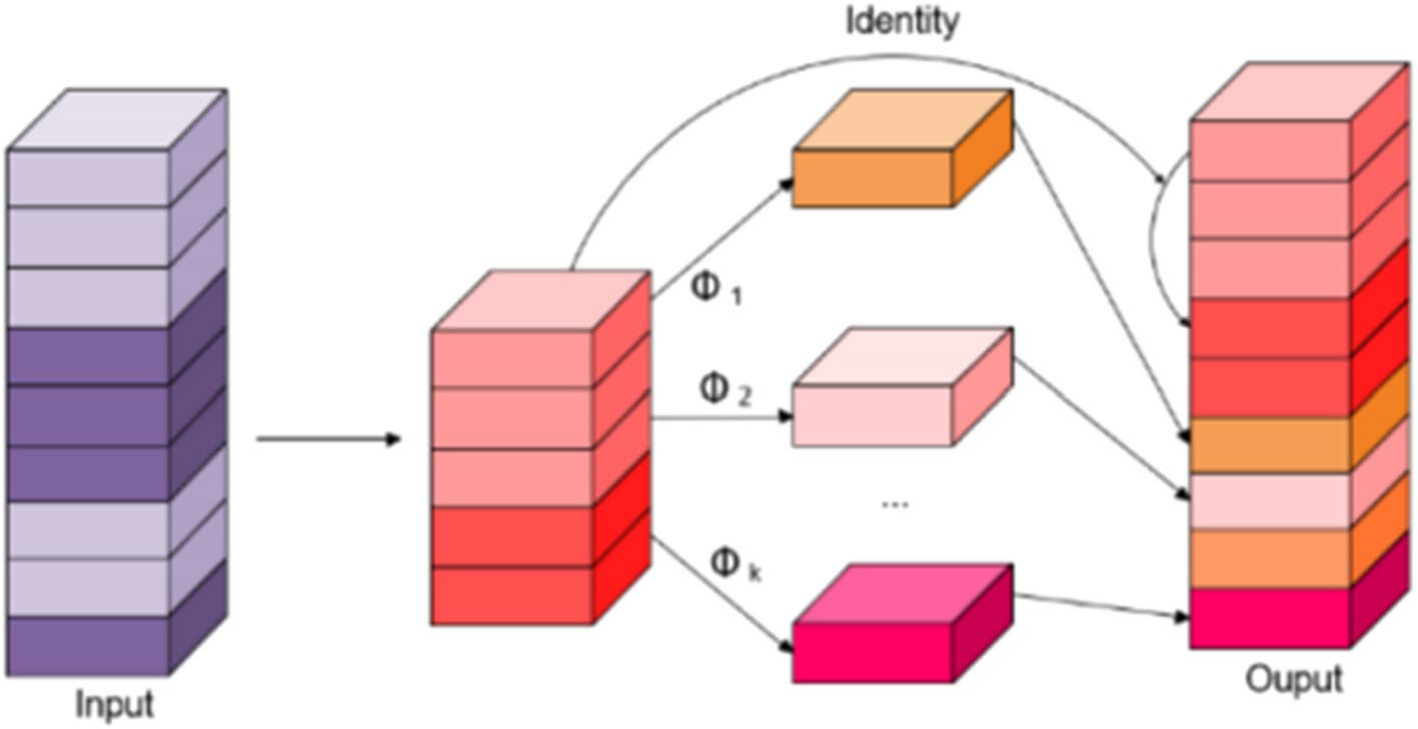
Structure of ghostConv.

The rapid inspection of foreign objects in power transmission lines is crucial for ensuring line stability. The traditional YOLOV8 algorithm, which employs the CBS (cross-stage partial network) to extract characteristics, incurs significant computational load due to its extensive convolution operations. To mitigate this, our study introduces a streamlined approach using GhostConv^37^, which notably reduces computational requirements through linear computation, as depicted in Fig. 2.

The detail the improvement as follows: Initially, GhostConv employs a select number of convolutional kernels to create a basic set of feature maps, effectively minimizing convolution operations. Consider *X* as the input, denoted as *X* ∈ *R*∧ (*h *× *w *× *c*). Here, *f′* symbolizes the convolution operation, and Y′ is the resultant feature map set post-convolution, expressed as Y′ ∈ R∧ (h′ × w′ × m′). The symbols hand w denote the height and width of the map, respectively. The letters c and m signify the number of input channels and output channels, respectively. The term k represents the size of the convolution kernel. The precise calculation method is outlined in Equation (1):

1 $$ {\text{Y}}^{\prime } = {\text{X}} \times {\text{f}}^{\prime } $$

GhostConv then conducts linear operations on them feature maps, denoted by Y′, to generate *m* × *s* feature maps. This process substantially reduces computational complexity. Each feature map for individual channels is indicated by *y′*_i_, where Φ symbolizes a straightforward linear transformation. In our research, GhostConv is adeptly integrated into the core and neck layers of the YOLOv8n algorithm. This integration is aimed at optimizing convolution operations to diminish both computational load and parameter quantity, resulting in a more efficient and lightweight network model. The specific calculation process is detailed in Eq. (2):

2 $$ y_{i,j} = \phi_{i,j} (y^{\prime }_{i} ) $$

### Bidirectional feature pyramid network (BiFPN)

In transmission line foreign object detection, a key challenge is managing the varying scales of detection targets. While the YOLOv8 model uses the PAN-FAN structure for multi-layer feature fusion, it results in increased model parameters and computational complexity. There’s also a potential loss of deeper semantic information while preserving surface-level semantics. To tackle this, our study introduces the BiFPN technique^38^. BiFPN, utilizing a weighted bidirectional feature pyramid, significantly improves feature fusion efficiency.

**Figure 3:**
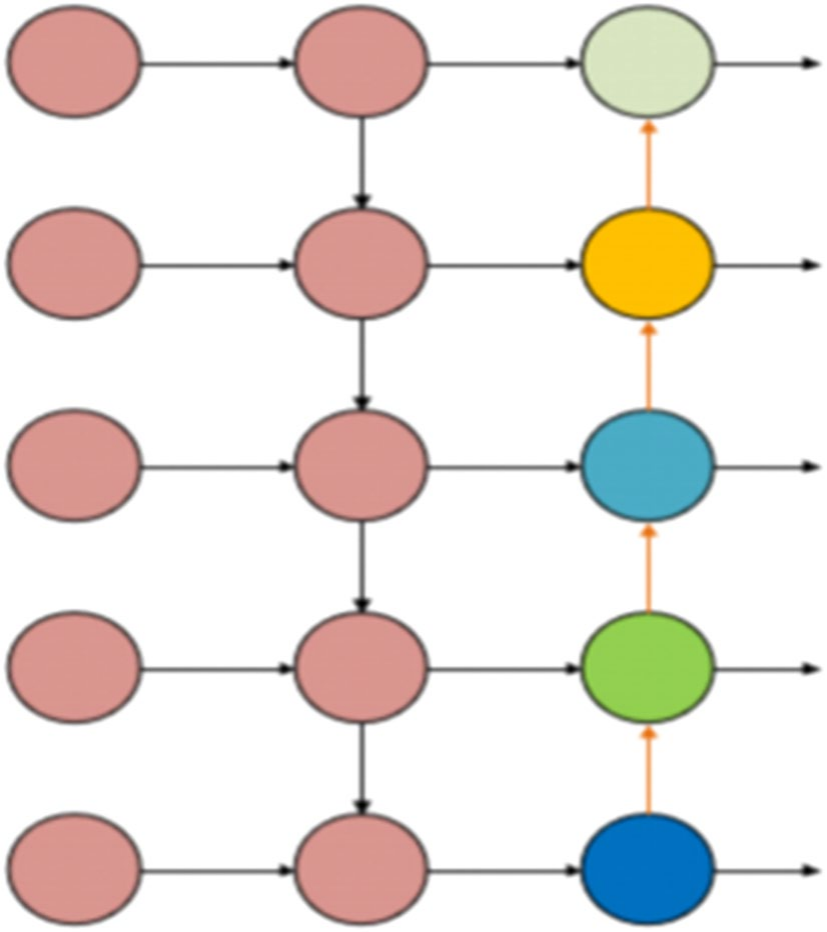
Design of PAN framework.

**Figure 4:**
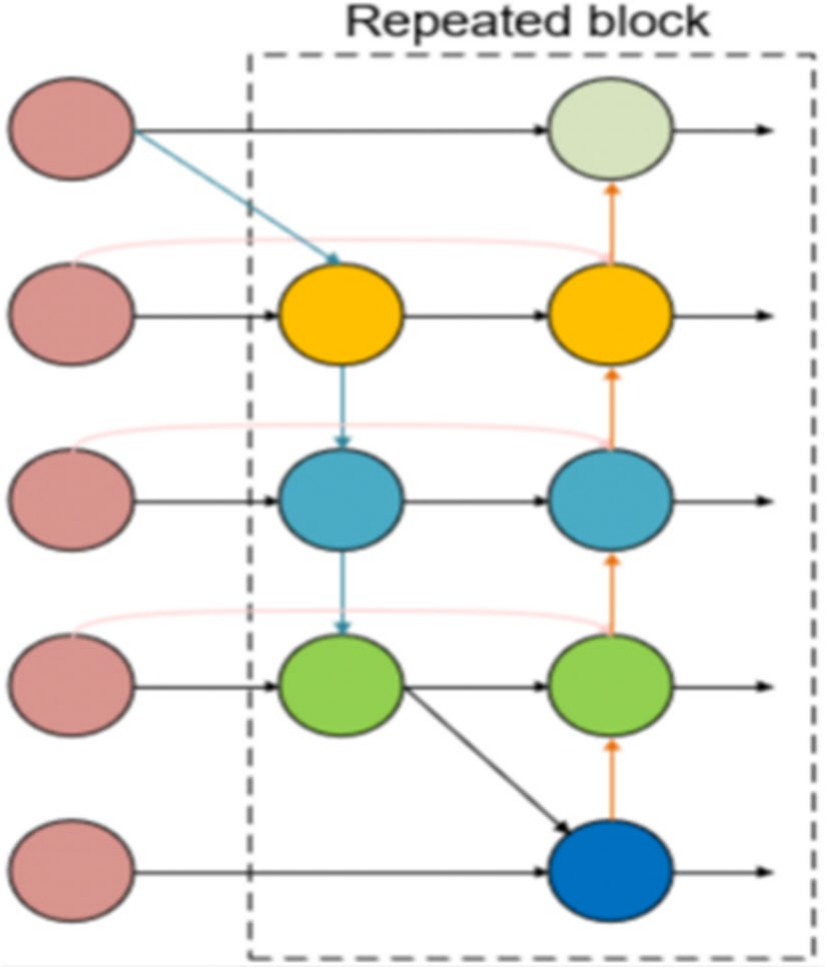
Design of BiFPN framework.

BiFPN’s initial step is to eliminate nodes that have a single input edge and limited contribution. This process substantially reduces the network’s complexity. Moreover, BiFPN boosts the efficiency of feature fusion for multi-scale targets by adding horizontal connections and performing repeated feature integrations. Detailed information is presented in Figs. 3, 4.

For optimal use of varying input features, BiFPN implements a rapid normalization method, assigning unique weights to each channel. The corresponding formula (3) is:

3 $$ O = \sum\limits_{i} {\frac{{w_{i} }}{{\varepsilon + \sum {_{j} w_{j} } }}} \cdot I_{i} $$

Here, *O* signifies the output, *I*_*i*_ is the input of the node, and *w*_*j*_ is the cumulative weight of the input nodes. For maintaining numerical stability, the learning rate is set at a minimum threshold of *e* = 0.0001.

### EC2f mechanism

**Figure 5:**
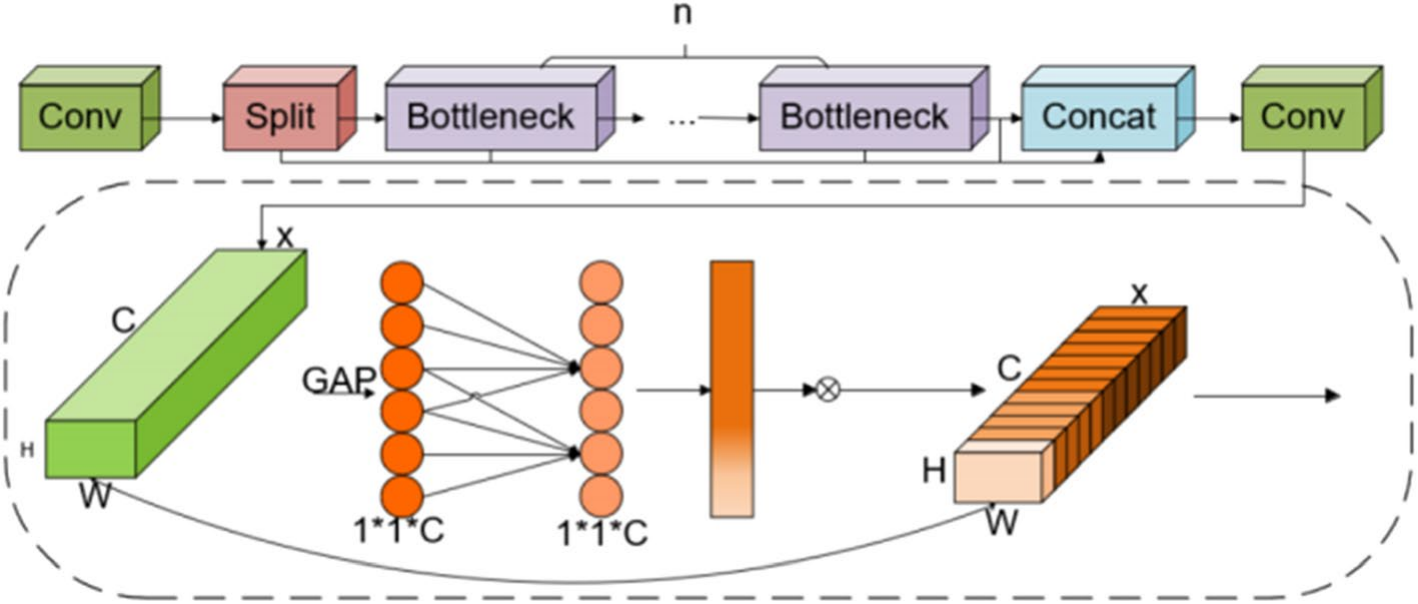
Design of EC2f. framework.

Extracting key features of foreign objects on transmission lines from complex backgrounds is a major challenge. The C2f module of YOLOv8 adopts multiple convolution components (Conv2d+BN+SiLU) and n BottleNecks, integrating low-level and high-level feature maps. It utilizes detailed and semantic information to improve the detection performance of targets of different scales. However, its utilization rate of output channels needs to be enhanced. Therefore, this paper proposes the EC2f mechanism to optimize the efficiency of feature fusion and the application of output features, as detailed in Fig. 5.

The ECA^39^ attention mechanism calculates attention weights in the channel dimension, unlike the positional dimension. It learns the importance of each channel through convolution operations, reducing computational complexity. Formula (4) defines the dimension k of the one-dimensional convolution kernel:

4 $$ {\text{k}} = \psi ({\text{C}}) = \left| {\frac{{\log_{2} {\text{C}}}}{\gamma } + \frac{{\text{b}}}{\gamma }} \right|_{odd} $$

where the nearest odd number to *t* is denoted by |*t* |*odd*.

In ECA attention mechanism, input features undergo global average pooling, transforming each channel’s feature map into a single value. This pooled feature map is then subjected to channel weight learning via a one-dimensional convolution layer. These learned weights are normalized and applied across each channel of the input feature map, resulting in a weighted feature representation. This process significantly improves the model’s proficiency in identifying vital inter-channel information.

## Experimental design and results 

### Dataset creation

Addressing the critical necessity of detecting foreign objects in power transmission lines is essential for reducing safety risks and maintaining stable operations. However, one of the primary challenges is the scarcity of relevant data in existing public datasets. To this end, this study utilized Baidu Maps and web crawler technology to successfully construct a dataset containing 1600 images. The images in this dataset were manually annotated using the LabelImg tool, with annotation information saved in TXT format files. To align with the needs of the experiment, the dataset included training, validation, and test sets, following a proportion of 8:1:1.

**Figure 6:**
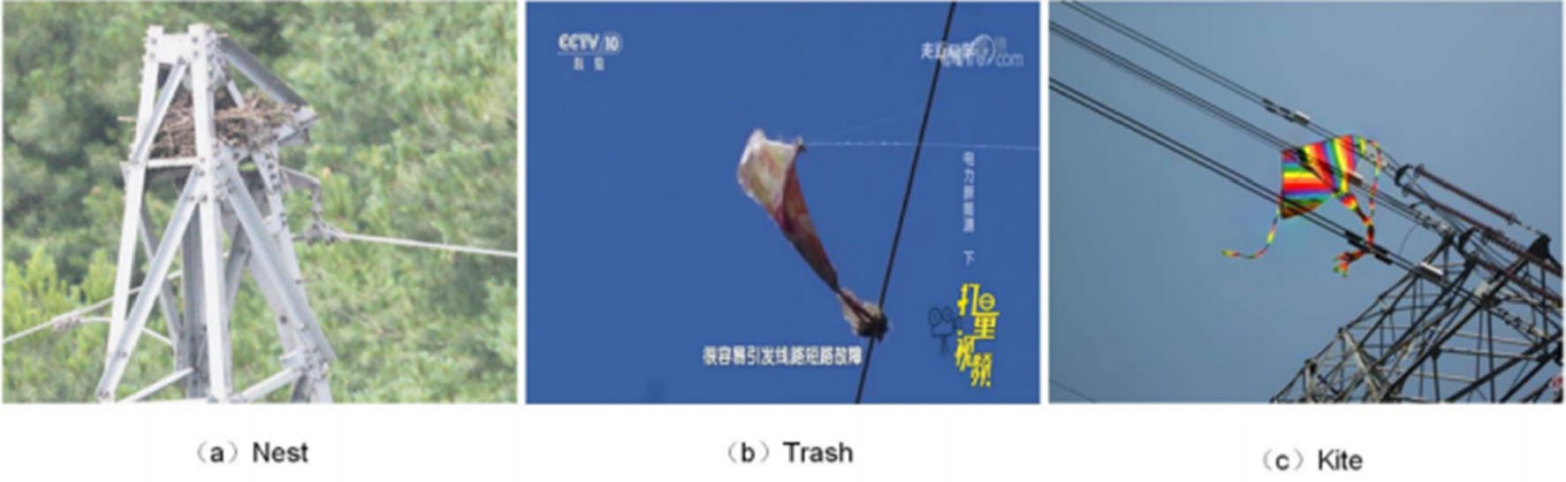
Three varieties of foreign objects.

Our dataset covers three varieties of foreign objects: nest, trash, and kites, with specific category distributions shown in Fig. 6.

**Figure 7:**
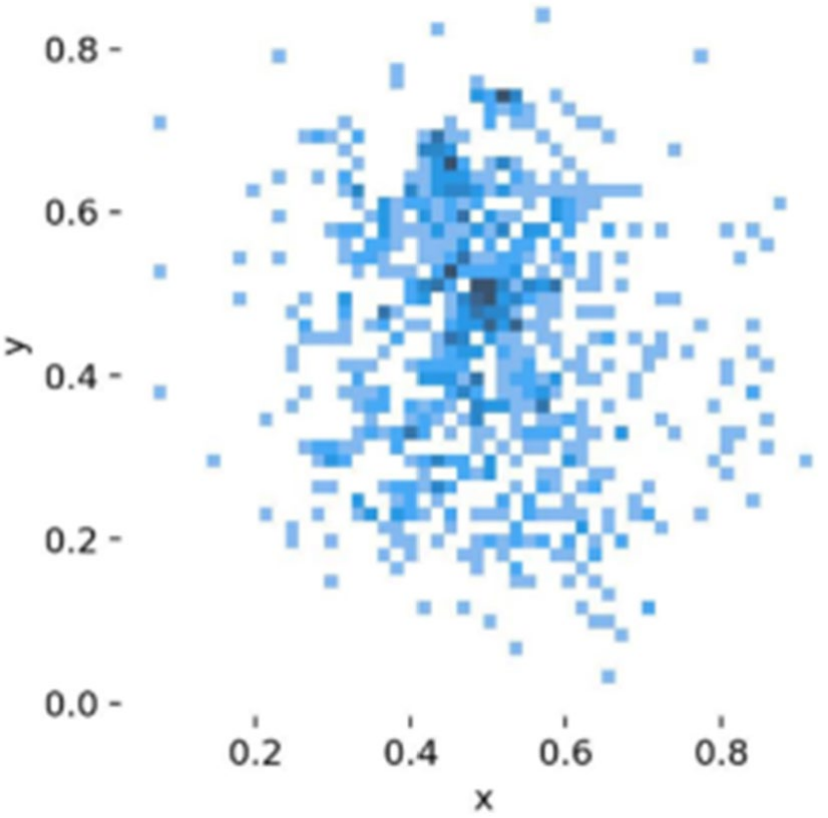
Central coordinates and size distribution of labeled boxes.

**Figure 8:**
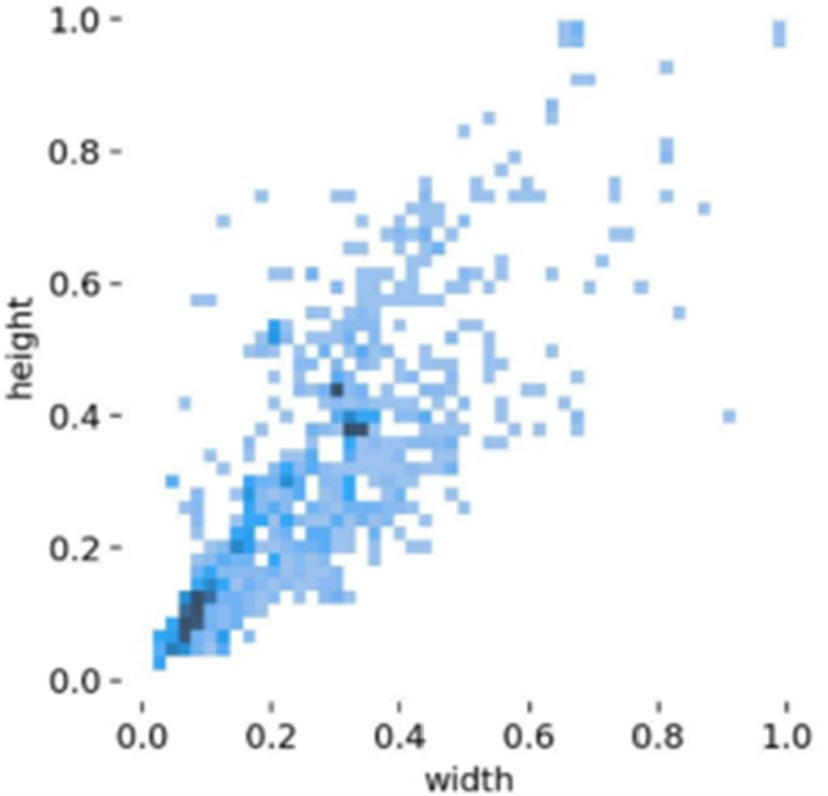
Length and width distribution of labeled boxes.

Figures 7, 8 display the data distribution and detailed labeling of the training dataset.

### Experimental setup and evaluation metrics

The environment consists of an Intel(R) Xeon(R) CPU E5-2680 v3 and an NVIDIA GeForce RTX 4090 graphics card (32GB video memory). A Linux system with Pytorch = 1.7.0 and Python = 3.8 was used. The experiment was run for 300 with a batch size of 32.

For bounding box-based object detection, metrics such as precision, recall, mean Average precision (mAP), and F1 are apt for evaluating the YOLO algorithm’s effectiveness^40–42^. These metrics provide a comprehensive view of an object detection algorithm’s accuracy, recall, and localization precision, offering a thorough assessment of the YOLO algorithm. The corresponding formulas are presented in Equations (5)–(8):

5 $$ P = \frac{TP}{{TP + FP}} $$

6 $$ R = \frac{TP}{{TP + FN}} $$

7 $$ F1 = \frac{{2 \times P^{ * } R}}{P + R} $$

8 $$ mAP = \frac{{\sum\nolimits_{q = 1}^{Q} {AP(q)} }}{Q} $$

where, precision represents the ratio of correctly predicted positive instances to all instances labeled positive by the model, indicating its accuracy in identifying positive cases. TP are correctly identified positive cases, whereas FP are negative cases incorrectly labeled as positive. Recall measures the fraction of actual positive cases correctly identified by the model. FN are positive cases misclassified as negative. The F1 score, the harmonic mean of precision and recall, evaluates the model’s overall accuracy and completeness.

Table 1 demonstrates GEB-YOLO’s superiority over YOLOv8n in transmission line foreign object detection, as evidenced by its enhanced precision, recall, F1, and mAP metrics, which increased by 3.7%, 5. 1%, 5.0%, and 6.8% respectively. The integration of the GhostConv module in GEB-YOLO has refined the network’s core structure, leading to a reduction in parameter count and a more streamlined network. The model also incorporates the EC2f attention mechanism. This mechanism employs a graduated approach from coarse to fine, thus improving the efficiency of information extraction in complex environments and enhancing precision in detection. Furthermore, the addition of the BiFPN module, known for its effective fusion capabilities, has improved the integration of multi-scale feature pyramids. This enhancement not only increases detection accuracy and efficiency but also effectively addresses challenges in multi-scale target detection. Overall, the comparative analysis of experimental outcomes indicates that GEB-YOLO excels beyond the YOLOv8n model in terms of detection efficiency.

**Table 1:** Comparative analysis of foreign object detection.

Model	Precision (%)	Recall (%)	F1 (%)	mAP (%)	Parameters/M
YOLOv8n	93.5	88.3	90.3	88.7	3.0
YOLO-LBS	97.2	93.4	95.3	95.5	2.7

**Figure 9:**
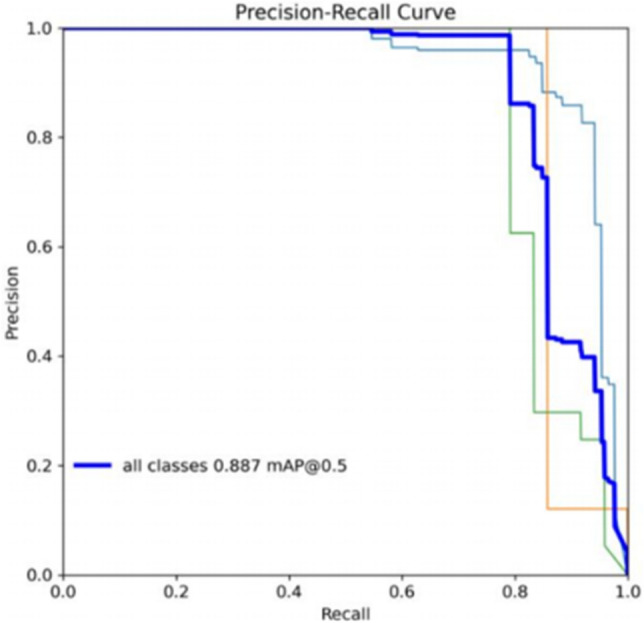
YOLOv8n’s precision-recall analysis.

**Figure 10:**
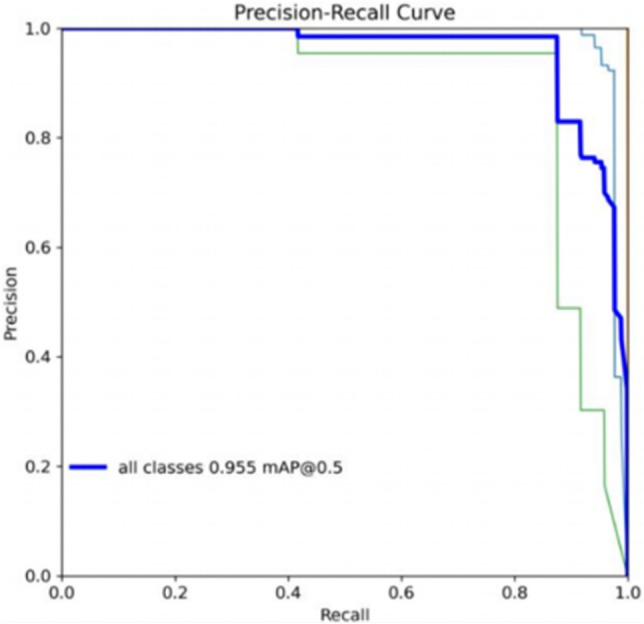
GEB-YOLOv8n’s precision-recall analysis.

Figures 9, 10 illustrate that the GEB-YOLO model’s precision-recall (P-R) curve encompasses a larger area compared to its sub-models. This area signifies the model’s mean average precision (mAP), with a larger area denoting enhanced detection performance. Consequently, the improved model exhibits markedly better detection capabilities than the YOLOv8n model.

### Ablation studies

**Figure 11:**
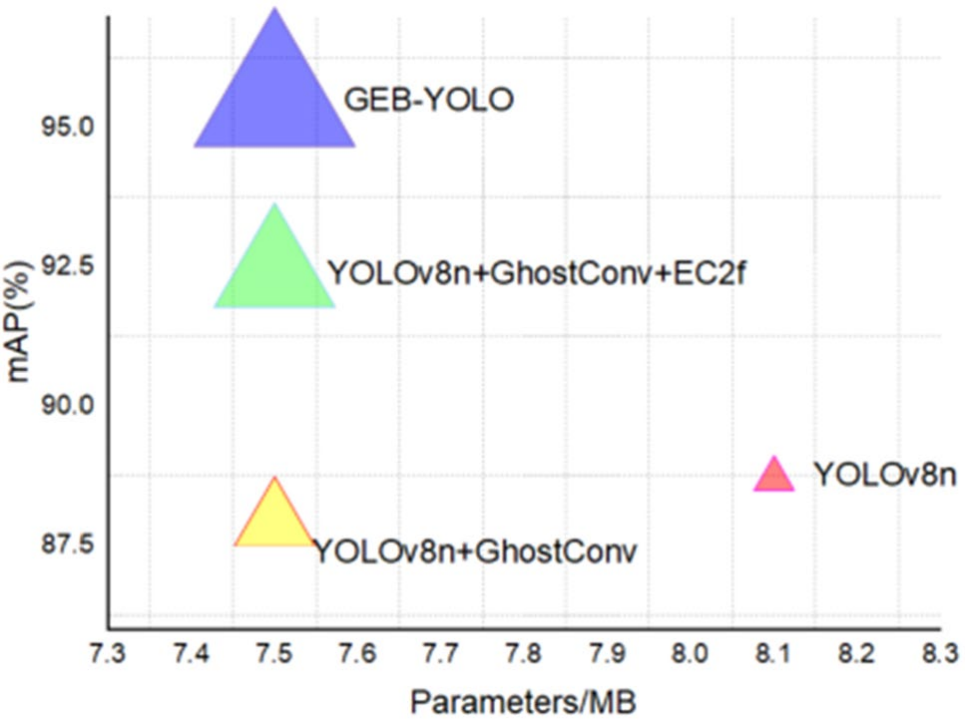
Comparative analysis of mAP across various model parameters.

This study delves into the impact of various improvement modules by using YOLOv8n as the baseline for ablation experiments. Table 2 and Fig. 11 clearly illustrate these impacts.

**Table 2:** Ablation study results.

Model	mAP (%)	Recall (%)	FLOPs/G	Parameters(M)
YOLOv8n	88.7	88.3	8.1	3.0
YOLOv8n + GhostConv	87.9	87.5	7.5	2.7
YOLOv8n + GhostConv + EC2f.	92.4	91.8	7.5	2.7
YOLOv8n + GhostConv + EC2f. + BiFPN (GEB-YOLO)	95.5	93.4	7.5	2.7

Referencing the findings presented in Table 2 and Fig. 11 reveal that each new module integration markedly boosts model performance. Notably, YOLOv8n + G registers a marginal decrease in mAP value compared to the YOLOv8n, attributed to the GhostConv module’s introduction. This module, designed to simplify convolution operations, effectively reduces computational complexity and contributes to the network’s lightweight structure. In contrast, YOLOv8n + E + B exhibits enhancements in all metrics. This improvement stems from the BiFPN module’s efficient feature fusion mechanism, which facilitates better integration of feature layers and thus improves the model’s capability to detect various-sized targets in intricate environments. Additionally, GEB-YOLO outperforms YOLOv8n + E + B across all parameters, thanks to the EC2f attention mechanism. This mechanism refines channel weights, thereby increasing feature recognition precision and the precision of foreign object detection in power transmission lines.

### Experiments with other models

**Figure 12:**
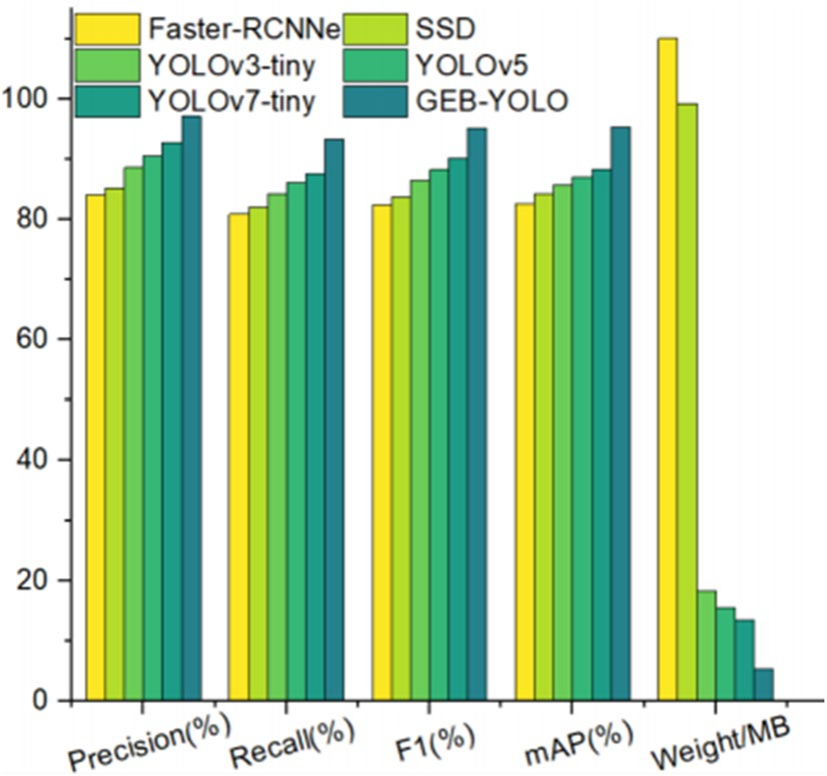
Study results with other models.

This research extends its analysis by benchmarking the GEB-YOLO model against prominent models utilizing an identical test set. The outcomes of these experiments are de-tailed in Table 3 and Fig. 12.

**Table 3:** Study results with other models.

Model	Precision (%)	Recall (%)	F1 (%)	mAP (%)	Weight/MB
Faster-RCNN	84.1	80.9	82.5	82.7	110.2
SSD	85.3	82.1	83.7	84.4	99.3
YOLOv3-tiny	88.7	84.4	86.5	85.8	18.4
YOLOv5	90.6	86.2	88.3	87.1	15.6
YOLOv7-tiny	92.9	87.6	90.2	88.3	13.5
GEB-YOLO	97.2	93.4	95.3	95.5	5.4

Data from these sources reveal that the GEB-YOLO model significantly outperforms others in terms of detection accuracy. While the Faster R-CNN model shows commendable accuracy, its complex network structure hinders its application in real-time detection. The SSD model, though computationally simpler, struggles with complex backgrounds and multi-scale targets. YOLOv3 offers improved multi-scale detection capabilities but at the cost of a larger structure and greater computational demands. YOLOv5, despite its high accuracy, has potential for further performance enhancements. YOLOv7, apt for real-time object detection, falls short in extracting features from targets of varying scales. The GEB-YOLO model’s experimental outcomes underscore its dual superiority in detection accuracy and model efficiency, outstripping the performances of its counterparts.

**Figure 13:**
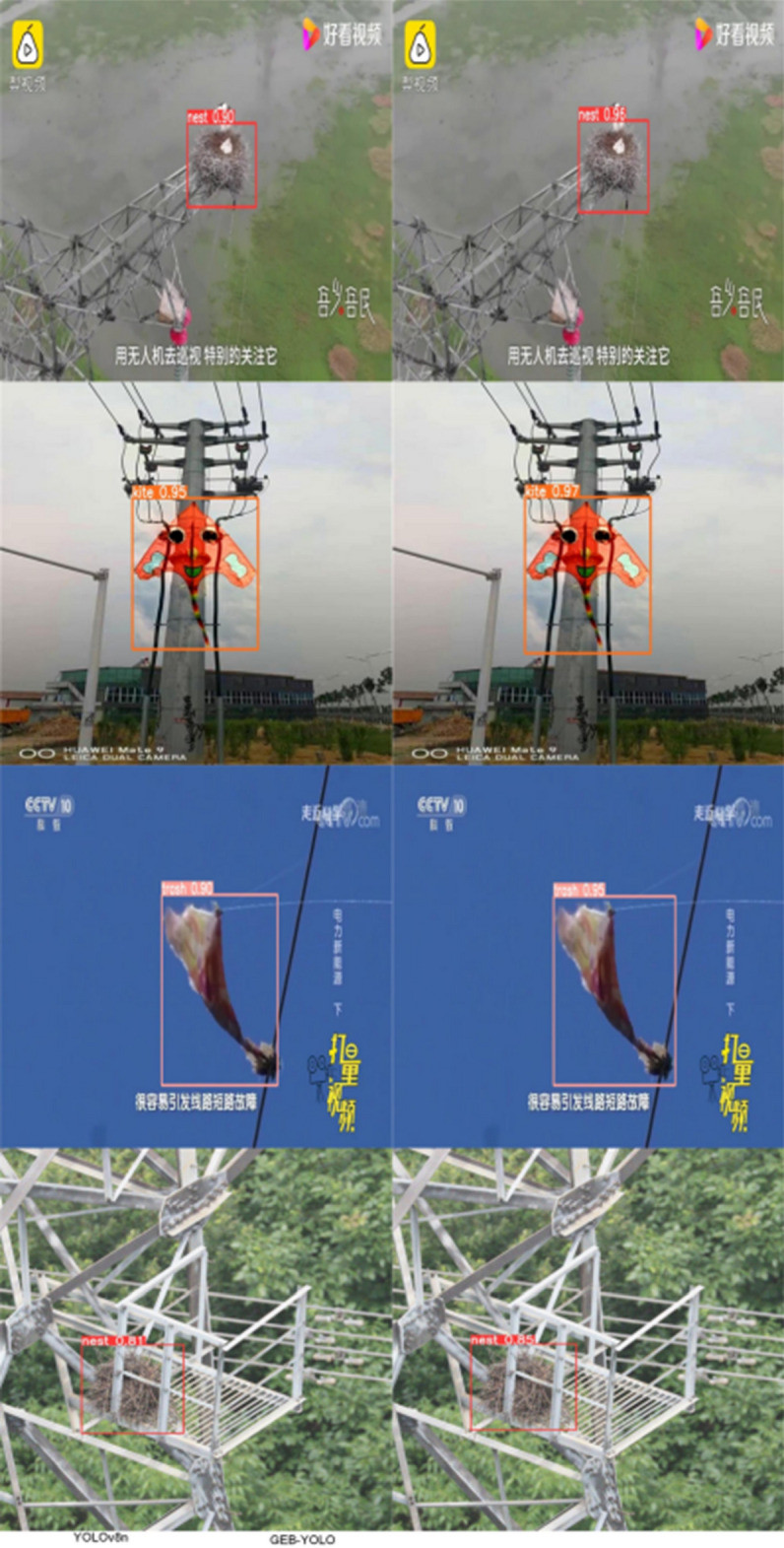
Testing outcomes for three distinct foreign objects.

This study’s optimized model underwent additional validation through the evaluation of selected images from the dataset. The analysis outcomes are presented in Fig. 13. These findings reveal that the proposed model demonstrates enhanced detection efficiency.

## Discussion

This study introduces the innovative GEB-YOLO model, aimed at enhancing safety and ensuring the stable operation of power transmission lines. The model is notable for its lightweight design and improved performance in detecting targets of various sizes.

Regarding feature extraction, the YOLOv8 model excels in the YOLO series but struggles with multi-scale targets due to its size. To address this, the study employs the GhostConv module’s efficient design to produce more detailed Ghost feature maps with reduced computational resources. This approach not only maintains high performance but also cuts down on computational costs. Additionally, the integration of the BiFPN module bolsters the feature pyramid’s ability to fuse information across different scales, thereby enhancing detection of multi-size targets. The paper further introduces the EC2f attention mechanism, which refines channel-level attention and enriches feature extraction, consequently enhancing the model’s overall performance.

This research introduces the GEB-YOLO model, an innovative deep learning-based approach, which outperforms previous methods inefficiently and accurately detecting foreign objects on power transmission lines. Table 3 illustrates that traditional deep learning dual-stage target detection models carry the drawback of larger model weights, a result of their complex network structures. Moreover, current single-stage object detection models show room for further improvement in their ability to detect foreign objects on power transmission lines. Our GEB-YOLO model, introduced in this research, significantly enhances this detection capability, making it particularly effective for identifying foreign objects on power transmission lines.

However, the GEB-YOLO model faces challenges in optimization and dataset comprehensiveness. For instance, while the GhostConv module minimizes computational efforts, it may inadvertently produce redundant information, impacting accuracy. Furthermore, the current dataset’s scope is limited and does not encompass all possible scenarios, thus restricting the model’s generalizability.

Future research will concentrate on dataset expansion and model optimization. Strategies include employing data augmenta- tion to gather a more diverse range of samples, thereby enhancing the model’s adaptability in complex environments. Moreover, the exploration of innovative training methodologies, optimization of loss functions, and the development of advanced feature generation techniques are planned to enhance the model’s efficacy and generalizability. These advancements are expected to notably ebhance the detection of foreign objects in power transmission lines.

## Conclusions

This research introduces the GEB-YOLO algorithm, designed to enhance the detection of foreign objects in power transmission lines, thereby improving safety and stability. This algorithm merges the lightweight Ghostconv network with the advanced YOLOv8, effectively reducing the load on computational and parameter resources. The introduction of the EC2f and BiFPN mechanisms substantially improves the model’s efficiency in processing various target sizes and extracting pertinent information. Experimental results reveal a marked improvement in detection performance over existing models. However, the current dataset does not include scenarios with adverse weather conditions. Future efforts will aim to broaden the dataset’s scope and further refine the algorithm for easier hardware implementation.

## Data Availability

The data employed in this research are available upon request from the corresponding authors.
